# The Performance of Paralleling Technique and Bisecting Angle Technique for Taking Periapical Radiographs: A Systematic Review

**DOI:** 10.3390/dj11070155

**Published:** 2023-06-21

**Authors:** Matthew Yen, Andy Wai Kan Yeung

**Affiliations:** 1 Faculty of Dentistry, University of Toronto, Toronto, ON M5S 1A8, Canada; matthew.yen@mail.utoronto.ca; 2 Oral and Maxillofacial Radiology, Applied Oral Sciences and Community Dental Care, Faculty of Dentistry, The University of Hong Kong, Hong Kong 999077, China

**Keywords:** dental medicine, diagnostic value, endodontics, periapical radiograph, radiology, systematic review

## Abstract

Periapical radiography is a routine radiographic procedure performed by dentists on a daily basis. It can be taken with two techniques, the paralleling technique (P tech) and the bisecting angle technique (B tech). This systematic review aimed to identify the relevant literature, compare the use of P and B techs across various dental specialties, and determine the most appropriate technique to be used for different purposes in taking periapical radiographs. In January 2023, we searched PubMed, Web of Science, Scopus, and Google Scholar to identify the studies that compared the two radiographic techniques. The search string was: (paralleling AND (“bisecting angle” OR “bisected angle”)). Manual reference tracing was also performed to identify the studies potentially missed. After screening, 26 studies were included for the qualitative review. The 26 included studies were published between 1976 and 2021. Ten of the studies were about general dentistry (dental radiology in general applications), whereas another ten studies were related to endodontics, such as working length estimation. Most studies advocated the use of the P tech for general, endodontics, implantology, and other indications. B tech was advocated for patients with a low palatal height. More future studies are needed to evaluate their performance in different scenarios with standardized equipment and radiographic positioning.

## 1. Introduction

Intraoral periapical radiographs are integral tools used to visualize the teeth and the surrounding structures. Proper periapical radiographs allow dental professionals to examine oral structures and associated pathologies with great accuracy to inform diagnosis, treatment planning, and the evaluation of prognosis. Taking periapical radiographs is a routine procedure performed by dentists almost on a daily basis, and there are many online videos providing educational information on the radiographic procedures related to this imaging modality [1]. Since it is frequently taken, it is important to ensure its diagnostic value to minimize the image reject rate or retake rate. A recent systematic review reported that the average reject rate of periapical radiography was approximately 16.4% [2]. Digital intraoral radiography is critical for providing comprehensive dental care and has gradually become the mainstream in dental clinics for many different purposes [3].

**Figure 1:**
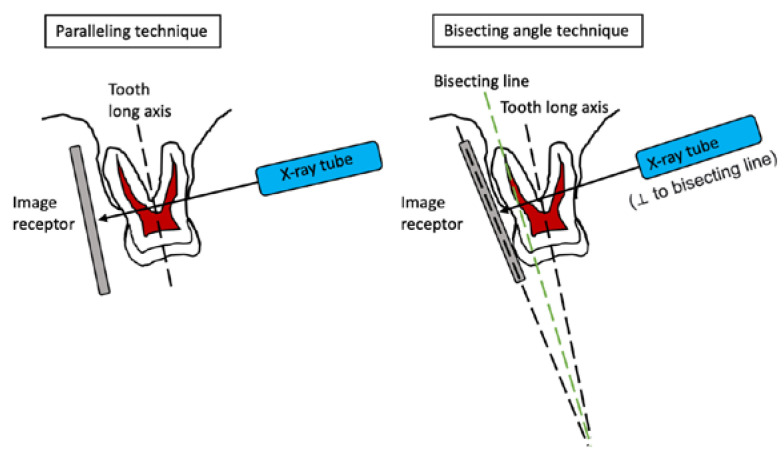
Schematic diagram illustrating the paralleling and bisecting angle techniques.

There are two radiographic techniques for taking periapicals: the paralleling technique (P tech) and bisecting angle technique (B tech) (Figure 1). P tech involves placing the film receptor parallel to the long axis of the target tooth such that the central x-ray beam is directed perpendicular to both the receptor and the tooth. The common notion, derived from earlier works such as [4], is that P tech is more reproducible and produces less distortion. Meanwhile, B tech can be more comfortable and it involves positioning the film receptor as close to the lingual (or palatal) side of the target tooth as possible, such that the central x-ray beam is directed perpendicular to an imaginary line that bisects the angle formed between the long axis of the tooth and the receptor. Indeed, a recent study reported that the P tech may not be applied well in the Asian population due to insufficient space in the upper maxilla region [5].

It is of paramount importance to understand the differences between the two techniques, and to seek evidence from the existing literature to recognize which of them should be used for various indications for optimized outcomes. To the best of the authors’ knowledge, there has been no systematic review on studies that compared the use of these two techniques. The authors were able to identify one review paper that covered both techniques of periapical radiography regarding their principle, instruments, technique, advantages, and disadvantages [6]; however, it did not report any comparisons between the two techniques based on the results of published studies. The current literature should have accumulated numerous reports that examined the two techniques for specific indications. This present study aimed to compare the use of P and B techs across various dental specialties and determine the most appropriate technique to be used for different purposes in taking periapical radiographs.

## 2. Materials and Methods

**Figure 2:**
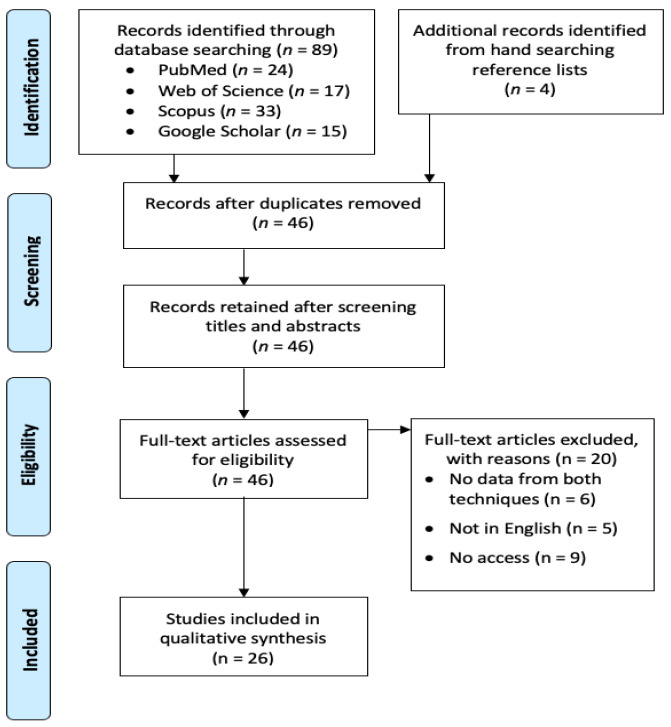
PRISMA flowchart of the screening process.

The study protocol was registered at PROSPERO (registration number: CRD42023383765). This study followed PRISMA guidelines. The following review aspects were considered. The review question was which of the P and B techs was most appropriate for various purposes in taking periapical radiographs. In terms of PICO, the patient type (P) could be any patient or extracted teeth; intervention (I) should be taking periapical radiographs; comparison (C) should be made between P and B techs; and outcome (O) should be in numerical values that reflected the performance of the radiographic techniques. In January 2023, we searched PubMed, Web of Science, Scopus, and Google Scholar to identify studies that compared the two radiographic techniques. The search string was: (paralleling AND (“bisecting angle” OR “bisected angle”)). No MeSH terms were added to the search because none of them were specific enough to cover this aspect of dental and maxillofacial radiology. The search fields were limited to the title and abstract for PubMed; the title, abstract, and keywords for Web of Science and Scopus; and the title for Google Scholar (using the advanced search function). Manual reference tracing was also performed to identify the studies potentially missed. A total of 93 studies were initially identified after the initial search strategy. After manually removing duplicates by checking the paper title and authorship, 46 studies remained. The inclusion criteria were original articles written in English. These 46 studies were all initially included and screened. They were excluded if they met any of the following exclusion criteria: did not report data from both radiographic techniques (regardless of on real patients or on specimens) for comparison (*n* = 6), not written in English (*n* = 5), or if we had no access to the full text (*n* = 9). Finally, 26 studies were included for the qualitative review. Two independent authors screened the literature and selected the studies. Disagreements were resolved by mutual discussion. The risk of bias assessment was performed with the NIH Quality Assessment checklist (https://www.nhlbi.nih.gov/health-topics/study-quality-assessment-tools (accessed on 6 April 2023)). Figure 2 shows a PRISMA flowchart of the screening process.

The authors did not contact the previous authors of relevant studies to ask for more studies or data, and did not search grey literature (i.e., unpublished papers or papers not indexed by the four databases used in this study).

For each study, we extracted the following information: the journal to be published, the country in which the work was performed, the indication/study purpose, the dental specialty concerned, the sample size, the statistical tests conducted, and the key findings.

## 3. Results and Discussion

The 26 included studies were published between 1976 and 2021. Many studies were from Asia, such as India (*n* = 5) and Malaysia (*n* = 3). Europe also contributed many studies, such as Norway (*n* = 5) and the United Kingdom (*n* = 4). The United States had two studies, whereas other countries contributed once each (Table 1). Ten of the studies were about general dentistry (dental radiology in general applications), whereas another ten studies were related to endodontics, such as working length estimation (Table 1). Two studies related to implantology, and forensics, orthodontics, pediatrics, and periodontics were investigated in one paper each.

Since the periapical radiograph can visualize the entire tooth up to the root apices and the peri-radicular region, it is intuitively suitable for many indications in endodontics that require the complete visualization of the root system, even for pediatric patients [32]. Indeed, there was a wide range of scope of these endodontic studies, which included the assessment of radiographic quality and accuracy of working lengths, root fractures, and periapical radiolucency lesion sizes. Ibrahim et al. [15] found that B tech had a higher retake rate (24.2%) than P tech (10.2%) in working length radiographs (*p* = 0.01). P tech was associated with lesser procedural errors than B tech in the maxillary arch (*p* < 0.05) and no significant difference was found between the techniques in the mandibular arch (*p* > 0.05). The study by Kazzi et al. [18] concluded that P tech had significantly lower diagnostic unacceptability rate (16.7%) than B tech (48.6%) (*p* < 0.001). Specifically, P tech produced significantly fewer errors in vertical angulation, cone cutting, and film placement (*p* < 0.001). The three studies by Forsberg [27,28,29] relevant to working lengths and root filling length all concluded that P tech was more accurate by producing fewer images with a >1 mm measurement deviation from the reference gold-standard images.

For measuring the actual tooth length, the study conducted by Abdul Razak and Abdul Razak [30] found that P tech was more accurate by producing a lower mean difference to the actual tooth length than B tech (+1.4 mm vs. +2.2 mm) (*p* < 0.001). In examining the root fractures, Iikubo et al. [14] found the effectiveness of the radiographic technique was dependent on the angle of the root fracture. The P tech was more sensitive to fractures at a 75-degree angle and at the long axis of the tooth, whereas the B tech was more sensitive to 55-degree angle fractures (*p* < 0.01). For measuring the periapical radiolucencies’ lesion size, one study by Forsberg and Halse [24] suggested that P tech reproduced fewer radiographs with a reduced periapical lesion size than B tech (2% vs. 10–20%) (*p* < 0.05). As the vertical angulation of the central beam increased, the error of lesion size detection increased for both P tech and B tech. In a later study, the same team [23] reported that there was no significant difference between the periapical lesion sizes as detected by P tech and B tech (*p* > 0.05).

In summary, almost all of the studies relevant to the endodontics preferred P tech. B tech was only preferred for examining root fractures that are at a 55-degree angle to the long axis of the tooth. While one study suggests P tech is superior for detecting the periapical radiolucency lesion size, another study concludes no significant difference between the techniques.

Meanwhile, 10 studies compared the use of P tech and B tech in the context of general dental practice. The scope of these studies included investigations into the radiographic error rate, radiation dose, comfort for patients, and practice/knowledge of dental professionals. Two studies examined the radiographic error rates. The study by Azizah et al. [13] reported the fewest radiographic errors when B tech was used with an external marker when compared to P tech and B tech used without an external marker. P tech committed 37 errors, B tech committed 45, and B tech with an external marker committed 31. There was a statistical difference between B tech and B tech with marker (*p* = 0.027), but no statistical difference between P tech and B tech (*p* = 0.206) or B tech with marker (*p* = 0.337). Meanwhile, the study by Mourshed and McKinney [31] showed that P tech when used with the Xtension Cone Paralleling Instrument (XCP) and the Precision X-ray instrument (Precision) had a lower retake rate than B tech. The retake rate was 23.1% for XCP, 22.8% for Precision, and 27.5% for B tech. The errors associated with incorrect vertical angulation and cone cutting were drastically lower in P tech than B tech (1.1–2.6% vs. 11.8%; 1.0–0.7% vs. 10.2%, respectively), but P tech had a higher rate of improper film positioning than B tech (25.6–28.4% vs. 18.5%). Three studies investigated the radiography practice and the knowledge of dental professionals from different countries. The study conducted by Aps [17] reviewed the questionnaire results by Flemish general dentists. Out of 374 valid questionnaires, 81% worked with P tech, 14% with B tech, and 5% did not know which technique they were using. However, the question on the questionnaire only asked about the paralleling technique instead of a choice between the paralleling and bisecting angle technique. This likely guided many respondents to answer affirmatively, which could potentially result in biased findings. In addition, Tugnait et al. [21] examined the practice and knowledge of dentists in England and Wales. Based on 592 valid questionnaire responses from GDPs, 31% always used P tech for periapical radiographs and 22% always used B tech. The use of P tech was higher among younger dentists and GDPs with additional postgraduate qualifications (*p* < 0.001). The study performed by Chandler and Koshy [22] examined approaches to radiography for root canal treatment by New Zealand dentists. A total of 931 valid responses revealed that both techniques were used regularly by GDPs and specialists, whereby P tech was used slightly more (26.3%) than B tech (22.4%).

Table 2 summarizes the preferred technique according to various important practical considerations. P tech had more superior performance over B tech in many aspects, except that the image retake rate was higher for P tech among patients with a low palatal height. Even so, it should be noted that the reported comfort level was at a medium range for both techniques without much difference. In fact, the P tech was the preferred technique for multiple indications, including detecting root fractures and implant misfits; measuring the length of a tooth, root, and/or crown–root ratio; and measuring the periapical lesion size. It was the more preferred technique in all but one survey. P tech was also found to have better performance in measuring the bone level for digital subtraction radiography and visualizing the incisive foramen. It had a lower radiation dose absorbed by the thyroid gland, and had a lower image reject rate in general. Meanwhile, the B tech was indicated in low palatal height patients and was equally error free when used with an external marker. There were two studies that reported no difference between the two techniques: one demonstrated no statistical difference between the techniques for the extent of root length distortion of maxillary deciduous molar roots, whereas the other one indicated no statistical difference for the periapical radiolucency lesion size assessment.

The risk of bias assessment is summarized in Table 3 and Table 4. Most of the studies were good quality case series, except that many of them did not explicitly mention if consecutive cases were examined. Half of the four survey studies reported an >50% response rate with sample size calculation, and were deemed good quality studies. Meanwhile, if the studies were considered in terms of the Oxford Centre for Evidence-Based Medicine 2011 Levels of Evidence, then all of them belonged to Level 3 (non-randomized studies) and Level 4 (case series and case–control studies).

Upon careful evaluation of the surveyed studies, we noticed an issue about the incongruent/non-standardized use of the P tech and B tech, with some studies including the use of intraoral grid and external markers in addition to the different vertical/horizontal angulations of the primary x-ray beam. These might complicate the applicability of the results, as clinicians might not have the same practice or non-standardized equipment in their clinics. Readers should note that the adjustment in the vertical angulation of the primary x-ray beam was often referred as a modification, such that the standard paralleling technique became a “modified” paralleling technique. This is frequently applied in clinical settings to overcome anatomical constraints such as in children [12]. Meanwhile, some studies examined all teeth, while others examined a specific subset of teeth. The findings from studies that investigated all teeth should theoretically be more generalizable than the findings from studies that investigated only a subset of teeth. In addition, most included studies examined the radiographic techniques applicable to general dentistry and endodontics, yet there was limited research in other specialties such as implantology, periodontics, orthodontics, pediatrics, oral surgery, and prosthodontics. It could be expected that the combination of B tech with direct intraoral sensors might be more prone to errors and retakes. Thus, much more research works are needed for these specialties, where periapical radiographs need to be taken more frequently.

The strength of this review was that it involved the searching of four major literature databases as well as hand searching. At the same time, this review had several limitations. First, there was a difficulty in accessing the literature, with nine articles without full access and five articles written in non-English. In addition, the literature databases might have inadequate coverage for older papers. The limited access to studies might have contributed to a limited scope of this review. Further, some studies did not disclose patient demographics, such as age and sex, so that there was a possibility for selection bias, or limited generalizability for the use of particular techniques in specific populations. Similarly, many of the surveyed studies had a relatively small sample size (fewer than 100 images per technique), therefore the data might not be readily generalized to the larger populations. The wide scope of this review encompassed the use of P tech and B tech in various specialties. However, the broad heterogeneity of studies contributed to inevitable variances in the equipment (such as film holders) and nuances (such as angulation) of the techniques, which might influence study results. The lack of uniformity between the studies, hence varying results, could make it difficult to precisely discern the strength of one technique over another. This made it easy to report study results individually, but made it difficult to compare them with tools such as confidence intervals (to determine precision). The wide variety of study outcomes makes standardized comparison difficult in conducting a meta-analysis. We recommended that future studies should recruit a larger sample size that can better represent the population, preferably determined based on sample size calculation. Another direction is to conduct multi-center studies so that the results can be more generalizable.

## 4. Conclusions

There were few studies on comparing the practical performance between the P tech and B tech, two radiographic techniques used to produce periapical radiographs. The authors could only identify 26 such studies published over the last 46 years. Most studies advocated the use of the P tech for general, endodontics, implantology, and other indications. B tech was advocated for patients with a low palatal height. Many studies were published during 1970s to 1990s that might seem outdated. More future studies are needed to evaluate their performance in different scenarios with standardized equipment and radiographic positioning in the modern setting.

## Figures and Tables

**Table 1:** Details of the 26 included studies.

Study	Journal	Country	Indication/Study Purpose	Dental Specialty	Sample Size	Statistical Tests	Key Findings
Veena et al., 2021 [7]	Journal of Morphological Sciences	India	Visibility of incisive foramen	Implantology	60 B tech/60 P tech	Chi-square test	Percentage of visibility on incisive foramen was higher in P tech than B tech (76.7% vs. 40.0%, *p* = 0.000)
Anand et al., 2020 [8]	European Journal of Molecular and Clinical Medicine	India	Opinion of techniques among dental professionals	General	250 dental students and dentists	N/A	B tech was chosen by most dental professionals (*p* < 0.05); equal preference for operator and patient comfort; P tech was favored for exposure parameters and image accuracy (*p* < 0.05)
Ahmad Satmi et al., 2020 [9]	Archives of Orofacial Sciences	Malaysia	Effectiveness and comfort of techniques in low palatal height patients	General	30 B tech/30 P tech	Mann–Whitney U test (for repeat rate)	Image repeat rate was higher in P tech than B tech (18.6% vs. 8.9%, *p* = 0.0251). Comfort was at medium range for both techniques
Reddy et al., 2019 [10]	Journal of Indian Academy of Oral Medicine and Radiology	India	Linear measurements with techniques, with conventional and digital film images, with the use of Intra Oral Grid	Endodontics, Periodontics	80/80/40/40 (conventional B tech, conventional P tech, digital B tech, digital P tech)	N/A	P tech showed a significant difference compared to B tech in conventional and digital methods with grid usage (*p* < 0.05)
Darós et al., 2018 [11]	Journal of Prosthetic Dentistry	Brazil	Diagnostic accuracy of techniques on misfit detection at the implant-abutment joint (IAJ)	Implantology	60 B tech/60 P tech/60 P tech with custom-made holder/60 bitewing	ROC curves (Az) and Fisher tests	P tech had significantly higher diagnostic values than B tech (*p* < 0.05) for 50- and 150-micron misfits
Sanghvi et al., 2018 [12]	Biomedical and Pharmacology Journal	India	Root length distortion of maxillary deciduous molar roots	Pediatrics	33 B tech/27 P tech	Chi-square test	No significant difference between techniques (*p* < 0.05)
Azizah et al., 2017 [13]	Medicine and Health	Malaysia	Radiographic error rate	General	80/80/80 (B tech, B tech with external marker and P tech)	Chi-square test	Number of radiographic errors was least in B tech with external marker (31), less in P tech (37) and highest in B tech (45) (B tech vs. P tech, *p* = 0.206; B tech vs. B tech with marker, *p* = 0.027)
Likubo et al., 2015 [14]	Oral Radiology	Japan	Diagnostic accuracy for root fractures	Endodontics	81 B tech/81 P tech	Kruskal–Wallis test	P tech had greater sensitivity for groove/fracture that were right-angles (*p* < 0.01) and 75-degrees to the long axis of the tooth (*p* < 0.05) than B tech, but B tech had greater sensitivity for the 55-degree groove (*p* < 0.01)
Ibrahim et al., 2013 [15]	Pakistan Oral	Pakistan	Reject rate in endodontic working length radiography	Endodontics	120 B tech/120 P tech	Chi-square test	B tech had a higher retake rate than P tech (24.2% vs. 10.2%, *p* = 0.01)
Kanchan-Talreja et al., 2012 [16]	Archives of Oral Biology	India	Accuracy of Kvaal formulae in Indian population for radiographic dental age estimation	Forensics	53 B tech/47 P tech	N/A	Average error of age estimation, for standard formula: P tech = ±18–20 years; B tech = ±19–21 years; for Indian-specific formula: both techs = ±11–14 years
Aps 2010 [17]	Dentomaxillofacial Radiology	Belgium	Practice and knowledge of dental radiography by Flemish general dentists	General	374 dentists	N/A	81% used P tech, 14% used B tech, 5% unclear
Kazzi et al., 2007 [18]	International Endodontic Journal	UK	Subjective image quality and radiographic errors of endodontic working length estimation films	Endodontics	37 B tech/60 P tech	Mann–Whitney U test	P tech had a significantly lower diagnostic unacceptability rate (16.7% vs. 48.6%, *p* < 0.001), incorrect vertical angulation rate (5.0% vs. 48.6%, *p* < 0.001) and cone cutting rate (20.0% vs. 62.2%, *p* < 0.001)
Rush and Thompson 2007 [19]	Radiography	UK	Radiation dose received at thyroid gland	General	32 B tech/32 P tech	ANOVA	Radiation dose received at thyroid gland was significantly lower in P tech than B tech (1.617 μGy vs. 4.863 μGy, *p* < 0.01)
Huh et al., 2005 [20]	Oral Surg, Oral Med, Oral Pathol, Oral Radiol, and Endo	Korea	Amount of error in alveolar crest level for digital subtraction radiography	Periodontics	360 B tech/360 P tech + biteblock/360 P tech	ANOVA	Amount of error was least in P tech with biteblock (0.108 mm), less in P tech (0.210 mm) and highest in B tech (0.277 mm) (*p* < 0.05). Error more severe in the molar region than in the anterior region
Tugnait et al., 2003 [21]	Journal of Dentistry	UK	Practice and knowledge of dental radiography by dentists in England and Wales	General	800 questionnaires (592 valid responses)	Chi-square test	31% used P tech, 22% used B tech; P tech was used more by younger dentists (*p* < 0.001)
Chandler and Koshy 2002 [22]	Dentomaxillofacial Radiology	New Zealand	Practice and knowledge of dental radiography for root canal treatment by New Zealand dentists	General	1200 questionnaires (931 valid responses)	N/A	26.3% used P tech, 22.4% used B tech; both techniques were in regular use
Forsberg and Halse 1997 [23]	International Endodontic Journal	Norway	Periapical radiolucencies’ lesion size	Endodontics	168 B tech/168 P tech	McNemar test	Detection of presence of lesions was same between techniques. Size of lesions was not significantly different (*p* > 0.05)
Forsberg and Halse 1994 [24]	International Endodontic Journal	Norway	Periapical radiolucencies’ lesion size	Endodontics	60 extracted teeth repeatedly imaged with multiple vertical angulations	N/A	P tech had lower ratio of cases with reduced lesion size than B tech (2% vs. 10–20%). Increased error as vertical angulation of central beam increased for both techs
Lecomber and Faulkner 1993 [25]	British Journal of Radiology	UK	Radiation dose absorbed by organs	General	14 B tech/4 P tech	N/A	P tech had lower radiation dose than B tech (e.g., upper molar: P tech = 1.51 μSv, B tech = 3.18 μSv)
Wood et al., 1989 [26]	Health Physics	Canada	Radiation dose absorbed by thyroid gland	General	20 B tech/20 P tech	T-test	P tech had lower absorbed dose at thyroid than B tech for 70-kVP beam (*p* < 0.05). However, P tech had higher dose at thyroid than B tech for 90-kVp beam (*p* < 0.05)
Forsberg 1987 [27]	International Endodontic Journal	Norway	Root filling length estimation	Endodontics	433 B tech/433 P tech	N/A	P tech (4%) had less images with >1 mm measurement deviation from the gold standard image for working length and root filling images than B tech (13–19%) when used with specially-constructed or Eggen film holder
Forsberg 1987 [28]	International Endodontic Journal	Norway	Root filling length estimation	Endodontics	200 B tech/200 P tech	N/A	With P tech as gold standard, B tech had >1 mm measurement deviation in 15% of cases (mostly shortened)
Forsberg 1987 [29]	Oral Surgery, Oral Medicine, Oral Pathology	Norway	Radiographic reproduction of working length	Endodontics	90 B tech/ 90 P tech	Chi-square test	P tech had >1 mm measurement deviation in 3–7% of cases; P tech was significantly more accurate at measuring the apical position of the metal indicator than B tech (*p* < 0.05)
Abdul Razak and Abdul Razak 1985 [30]	Dental Journal of Malaysia	Malaysia	Accuracy in measuring tooth length	Endodontics	120 B tech/120 P tech	N/A	The mean difference with the actual length was +1.4 mm for P tech and +2.2 mm for B tech (*p* < 0.001); B tech had a greater deviation range and was more magnified than P tech
Biggerstaff and Phillips 1976 [4]	Oral Surgery	US	Variability of measured crown–root ratio	Orthodontics	5 B tech/5 P tech	N/A	Variance of radiographic of crown–root ratio was less for P tech than B tech (0.000019 vs. 0.000894) (*p* = 0.0005)
Mourshed and McKinney 1972 [31]	Oral Surgery, Oral Medicine, Oral Pathology	US	Radiographic error rate	General	3236 P tech with XCP/2582 P tech with Precision/ 2670 B tech with Snap-A-Ray holder	N/A	B tech had greater retake rate (27.5% vs. 23.1–22.8%), incorrect vertical angulation (11.8% vs. 1.1–2.6%), and cone cutting (10.2% vs. 1.0–0.7%) than P tech; P tech had greater improper film positioning (25.6–28.4% vs. 18.5%)

**Table 2:** Preferred technique according to key practical considerations.

Key Practical Considerations/Clinical Indications	Preferred Technique	Supporting Studies
Diagnostic accuracy (e.g., root fractures, implant misfits)	P tech	[11,14]
Bone level measurement for digital subtraction radiography	P tech	[20]
Tooth length/root length/crown–root ratio estimation	P tech	[4,10,27,29,30]
	No difference	[12]
Periapical lesion size measurement	P tech	[24]
	No difference	[23]
Visibility of incisive foramen	P tech	[7]
Radiation dose absorbed by thyroid and other organs	P tech	[19,25,26]
Practice preference (from four survey studies)	P tech	[17,21,22]
	B tech	[8]
Radiographic error rate/reject rate	P tech	[13,15,18,31]
	B tech (for low palatal height)	[9]

**Table 3:** Risk of bias assessment of the 22 radiographic studies.

Study	1. Was the Study Question or Objective Clearly Stated?	2. Was the Study Population Clearly and Fully Described, including a Case Definition?	3. Were the Cases Consecutive?	4. Were the Subjects Comparable?	5. Was the Intervention Clearly Described?	6. Were the Outcome Measures Clearly Defined, Valid, Reliable, and Implemented Consistently across all Study Participants?	7. Was the Length of Follow-Up Adequate?	8. Were the Statistical Methods Well Described?	9. Were the Results Well Described?	Quality Rating (Good, Fair, or Poor)
Veena et al., 2021 [7]	Yes	Yes	NR	Yes	Yes	Yes	NA	Yes	Yes	Good
Ahmad Satmi et al., 2020 [9]	Yes	Yes	NR	Yes	Yes	Yes	NA	Yes	Yes	Good
Reddy et al., 2019 [10]	Yes	Yes	NR	Yes	Yes	Yes	NA	No	Yes	Good
Darós et al., 2018 [11]	Yes	Yes	NR	Yes	Yes	Yes	NA	Yes	Yes	Good
Sanghvi et al., 2018 [12]	Yes	Yes	NR	Yes	Yes	Yes	NA	Yes	Yes	Good
Azizah et al., 2017 [13]	Yes	Yes	NR	Yes	Yes	Yes	NA	Yes	Yes	Good
Likubo et al., 2015 [14]	Yes	Yes	NR	Yes	Yes	Yes	NA	Yes	Yes	Good
Ibrahim et al., 2013 [15]	Yes	Yes	NR	Yes	Yes	Yes	NA	Yes	Yes	Good
Kanchan-Talreja et al., 2012 [16]	Yes	Yes	NR	Yes	Yes	Yes	NA	Yes	Yes	Good
Kazzi et al., 2007 [18]	Yes	Yes	NR	Yes	Yes	Yes	NA	Yes	Yes	Good
Rush and Thompson 2007 [19]	Yes	Yes	NR	Yes	Yes	Yes	NA	Yes	Yes	Good
Huh et al., 2005 [20]	Yes	Yes	NR	Yes	Yes	Yes	NA	Yes	Yes	Good
Forsberg and Halse 1997 [23]	Yes	Yes	NR	Yes	Yes	Yes	Yes	Yes	Yes	Good
Forsberg and Halse 1994 [24]	Yes	Yes	NR	Yes	Yes	Yes	NA	No	Yes	Good
Lecomber and Faulkner 1993 [25]	Yes	Yes	NR	Yes	Yes	Yes	NA	No	Yes	Good
Wood et al., 1989 [26]	Yes	Yes	NR	Yes	Yes	Yes	NA	Yes	Yes	Good
Forsberg 1987 [27]	Yes	Yes	Yes	Yes	Yes	Yes	NA	No	Yes	Good
Forsberg 1987 [28]	Yes	Yes	NR	Yes	Yes	Yes	NA	No	Yes	Good
Forsberg 1987 [29]	Yes	Yes	NR	Yes	Yes	Yes	NA	No	Yes	Good
Abdul Razak and Abdul Razak 1985 [30]	Yes	Yes	NR	Yes	Yes	Yes	NA	No	Yes	Good
Biggerstaff and Phillips 1976 [4]	Yes	Yes	NR	Yes	Yes	Yes	NA	No	Yes	Good
Mourshed and McKinney 1972 [31]	Yes	Yes	NR	Yes	Yes	Yes	NA	No	Yes	Good

NA, not applicable. NR, not reported. Red, high risk of bias. Yellow, unclear risk of bias. Green, low risk of bias.

**Table 4:** Risk of bias assessment of the four survey studies.

Study	1. Was the Study Question or Objective Clearly Stated?	2. Was the Study Population Clearly Specified and Defined?	3. Was the Participation Rate of Eligible Persons at Least 50%?	Were all Subjects Recruited from the Same or Similar Populations? Were the Inclusion and Exclusion Criteria Applied Uniformly?	5. Was a Sample Size Justification, Power Description, or Variance and Effect Estimates Provided?	6. Were the Exposures of Interest Measured Prior to the Outcomes Being Measured?	7. Were the Outcome Measures Clearly Defined, Valid, Reliable, and Implemented Consistently?	8. Were Key Potential Confounding Variables Measured and Adjusted Statistically?	Quality Rating (Good, Fair, or Poor)
Anand et al., 2020 [8]	Yes	No	NR	Yes	No	Yes	Yes	No	Fair
Aps 2010 [17]	Yes	Yes	NR	Yes	No	Yes	Yes	Yes	Fair
Tugnait et al., 2003 [21]	Yes	Yes	Yes	Yes	Yes	Yes	Yes	Yes	Good
Chandler and Koshy 2002 [22]	Yes	Yes	Yes	Yes	Yes	Yes	Yes	Yes	Good

NA, not applicable. NR, not reported. Red, high risk of bias. Yellow, unclear risk of bias. Green, low risk of bias.

## Data Availability

Not applicable.
